# Telemedicine Technologies Selection for the Posthospital Patient Care Process after Total Hip Arthroplasty

**DOI:** 10.3390/ijerph191811521

**Published:** 2022-09-13

**Authors:** Karolina Kamecka, Calogero Foti, Łukasz Gawiński, Marek Matejun, Anna Rybarczyk-Szwajkowska, Marek Kiljański, Marek Krochmalski, Remigiusz Kozłowski, Michał Marczak

**Affiliations:** 1 Department of Management and Logistics in Healthcare, Medical University of Lodz, 90-131 Lodz, Poland; 2 Physical and Rehabilitation Medicine, Clinical Sciences and Translational Medicine Department, Tor Vergata University, 00133 Rome, Italy; 3 Department of Entrepreneurship and Industrial Policy, Faculty of Management, University of Lodz, 90-237 Lodz, Poland; 4 Polish Association of Physiotherapy Specialists, 95-200 Pabianice, Poland; 5 Medical Magnus Clinic, 90-552 Lodz, Poland; 6 Polish Muscles, Ligaments and Tendons Society, 90-552 Lodz, Poland; 7 Center of Security Technologies in Logistics, Faculty of Management, University of Lodz, 90-237 Lodz, Poland

**Keywords:** telehealth, telerehabilitation, teleconsultation, telemonitoring, visual telemedicine technologies, wearable telemedicine technologies, smart wearables, total hip arthroplasty, posthospital period, patient care

## Abstract

**Abstract:**

For many years, the importance of using telematic technologies in medicine has been growing, especially in the period of the coronavirus pandemic, when direct contact and supervision of medical personnel over the patient is difficult. The existing possibilities of modern information and communication technologies (ICTs) are not fully used. The aim of the study is to identify the telemedicine technologies that can be used in future implementation projects of the posthospital patient care process after total hip arthroplasty (THA). The literature search is reported according to PRISMA 2020. The search strategy included databases and gray literature. In total, 28 articles (EMBASE, PubMed, PEDro) and 24 records from gray literature (Google Search and Technology presentations) were included in the research. This multi-source study analyzes the possibilities of using different technologies useful in the patient care process. The conducted research resulted in defining visual and wearable types of telemedicine technologies for the original posthospital patient care process after THA. As the needs of stakeholders in the posthospital patient care process after THA differ, the awareness of appropriate technologies selection, information flow, and its management importance are prerequisites for effective posthospital patient care with the use of telemedicine technologies.

**Protocol Registration:**

PROSPERO 2022 #CRD42022320491

## 1. Introduction

Delivering healthcare services at a distance by using continuously developing information and communication technologies (ICTs) is one of the key challenges of our time. It is obviously also an extremely important aspect of the future of worldwide medicine. Telemedicine entered outpatient specialist care, hospitals, and patients’ homes [1,2]. Undoubtedly, over the last decade, scientists and clinical practitioners have already collected a lot of evidence that telemedicine has helped to solve a great number of problems resulting, inter alia, from epidemiological emergencies, inducing the requirement of social distance. Moreover, at the same time, it turned out to be a possible additional, acceptable, and effective form of helping patients in specific circumstances [3,4]. By being an application of telematics in medicine, telemedicine includes technologies aimed at collecting information, analyzing, sending, processing, presenting it, and sharing it with stakeholders.

Stakeholders (patients, physicians, nurses, and physiotherapists) have high expectations for the use of telematic technologies to improve the quality of health care service at a sustainable cost. Many countries within the Organization for Economic Cooperation and Development (OECD) are investing in telemedicine technologies, which encourages a growing body of peer-reviewed studies on the topic [5]. The process of implementing telemedicine solutions into clinical practice revealed new financial, technological, legal, IT, and awareness barriers. Highly developed countries, which are characterized by the biggest development of telemedicine systems, have introduced and are still introducing many solutions facilitating the use of such systems in practice [6].

The aim of the article is to identify telemedicine technologies that can be used in the already designed posthospital patient care process after total hip arthroplasty. This article is the next natural step on the way to pilot implementation of the process in the future. The proper selection of technologies is of key importance in modern technology management and will contribute to the successful use of the technology in the treatment process of patients.

**Figure 1:**
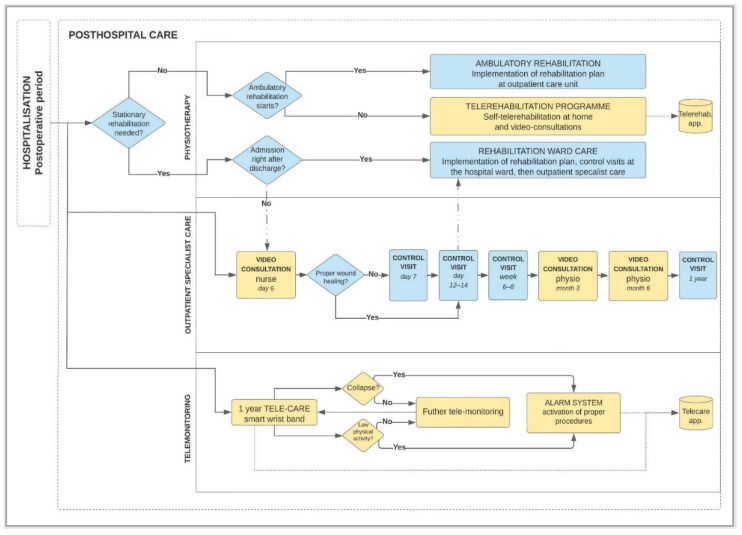
Diagram of posthospital patient care process after THA using telemedicine technologies.

The original process of posthospital patient care after total hip arthroplasty (THA) designed within InterDoktorMen project by Kamecka et al. [7] includes innovative aspects of modern telemedicine solutions use. The process stands as an innovative holistic proposal possible to be implemented in the near future in Poland. Before the study, the implementation required us to identify telemedicine technologies. The designed process includes a few areas of innovation where telemedicine solutions are needed within physiotherapy, outpatient specialist care, and patient telemonitoring (figures marked in yellow in Figure 1). The yellow fields that cover the three key telemedicine concepts are the subjects of interest in this article, namely:

- Teleconsultation,
- Telerehabilitation,
- Telemonitoring.

**Figure 2:**
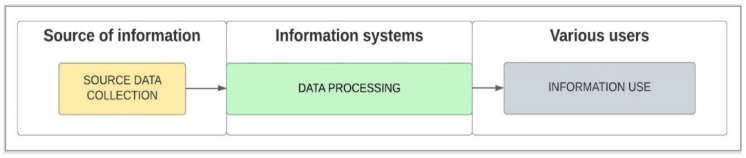
Diagram of information flow in posthospital process of patient care after THA using telemedicine technologies.

Because information plays a key role in the designed process, the preparation stage for the implementation requires characteristics of the information flow. The flow, in general, consists of source data collection, data processing via information systems, and final use of information by stakeholders (Figure 2).

Telemedicine solutions that are planned to be used in the specific process require adaptation to specific scenarios; therefore, we decided to conduct a detailed technology selection process, presented in the following parts of the article.

## 2. Materials and Methods

The stages of research were designed as follows: defining research questions, search strategy (literature and market analysis), data identification, screening, eligibility, selection, and narrative synthesis.

### 2.1. Research Questions

An interdisciplinary group of specialists conducting this research consists of representatives of various scientific disciplines, namely: Public Health, Health Management, Health Logistics, Process Management, Orthopedics, and Rehabilitation. The group defined the following research questions:

1. Who uses telemedicine technologies in the posthospital patient care process after THA?
2. What data are collected from telemedicine technologies used in the posthospital patient care process after THA?
3. What are the needs of stakeholders in the posthospital patient care process after THA?
4. What technologies are more common in the postoperative patient care process after total hip arthroplasty?
5. Will it be possible to define groups of the most important technologies used in the studied process?

### 2.2. Literature Search

Based on these research questions, the search strategy was designed to identify relevant literature regarding telemedicine solutions used in posthospital patient care after THA, with the main attention on used technologies and user groups. This literature search is reported according to the Preferred Reporting Items for Systematic Reviews and Meta-Analyses statement (PRISMA Statement [8]); the review has been registered with PROSPERO (International Prospective Register of Systematic Reviews) under the identifier #CRD42022320491. The search results were used to create a selection of telemedicine technologies, which is the subject of the study. The EMBASE, PubMed, and Physiotherapy Evidence Database (PEDro) databases were searched using the search terms: “ehealth”, “telehealth”, “telerehabilitation”, “telemonitoring”, “teleconsultation”, “telemedicine”, “videoconferencing”, “remote consultation”, “total hip arthroplasty”, “hip replacement”, “hip joint”, “hip osteoarthritis”, “postoperative care” (Supplementary Materials File S1).

Full-text articles that could potentially meet the inclusion criteria for the review were assessed by the authors against the inclusion criteria. To meet these inclusion criteria and to be eligible for the analysis, the studies had to meet the PICO-based eligibility criteria: The target population (P) studies with adult participants after THA were included. The studies in which participants’ care process was not related to THA (e.g., cardiac disorders, stroke, upper limb disability, pulmonary disorders, and Parkinson’s disease) were excluded. Intervention (I) was defined as the use of telemedicine technology in the posthospital patient care process after THA. Only studies where any telemedicine technology was used in postoperative patient care after THA in an experimental or observational study were included. The study intervention had to focus on patient care after hip replacement procedure, including telerehabilitation, teleconsultation, or telemonitoring in a year period after the surgery by different users taking part in the process. Insufficient technical explanation in searched studies was an exclusion criterion. For comparison (C), all trials were included, whether they did or did not employ a control group. The results (O) included the selection of telemedicine technologies used in postoperative patient care after THA, defining users and their needs in postoperative patient care after THA. Original English-language studies published from January 2012 to April 2022 were included when the eligibility criteria from our PICO framework were fulfilled. Books and Documents, Clinical Trials, Meta-Analyses, Randomized Controlled Trials, and Systematic Reviews were included. Review studies, abstracts, and conference papers were excluded. Studies having a population of patients after total hip replacement, targeting the postoperative period, and using telemedicine technology were included.

### 2.3. Technology Market Analysis

#### 2.3.1. Market Analysis Methods

The research task was to analyze the market in the field of telemedicine technologies available to retail users (patients) and medical staff aimed at supporting the process of patient care after THA. In the market analysis, the following sources of information were used: Google Search tool, practical experience of authors from a medical facility, as well as presentations and materials on new technologies provided by technology suppliers. All the mentioned sources constitute gray literature included in the research as in the PRISMA chart and are described in subsections as follows.

#### 2.3.2. Google Search

The Google Search tool was used in the study. The survey was carried out in May 2022. In order to find the most accurate answers to research questions, the following keywords were introduced to the advanced Google Search tool: “software for medical video consultation” OR “software for orthopedic telerehabilitation” OR “telemetric wrist band with collapse function” OR “telecare monitoring”. Additional search settings were: deletion of search history and cookies before starting the search, keywords search on the entire page, search period as last year, keywords in English (widely used global language) and in Polish (appropriate to be used in the locally analyzed process, important for local implementation of the process) were used.

Only websites displayed on the first three pages of the Google Search engine were searched and the content was analyzed. The aim of the first three pages was to (1) exclude the risk of selecting suppliers of technologies with poor implementation potential and (2) strengthen the search for identification of experienced technology suppliers, strong in terms of implementation and communication and with high development potential.

The analysis of the usefulness of searched telemedicine technologies in the designed process of posthospital patient care after THA was performed. The website addresses were noted, and the content of the pages was analyzed. A searched technological solution was included in the research if it fulfilled one or more following criteria:

- Technology is used in the healthcare sector, in specialistic medical programs, clinical trials, or EU co-financed grants,
- Technology meets the professional criteria, approvals, or recommendations recognized for telemedicine technologies by global organizations such as government institutions, the Food and Drug Administration (FDA), World Health Organization (WHO), American Telemedicine Association ATA), or medical device regulations (EU Medical Device Regulation MDR 2017/745) [9,10,11,12],
- Technology is mentioned in scientific publications over technology application,
- Technology or its direct supplier is certified in management or security systems or other specialist certifications specific to the health sector.

#### 2.3.3. Practical Experience and Technology Knowledge

The Medical Magnus Clinic was selected for the study due to the fact that it is a medical facility specializing in orthopedics and rehabilitation of the musculoskeletal system, including hip and knee arthroplasty procedures. In the period 2020–2021, the facility took first place among all National Health Fund (NFZ) service providers in terms of hip and knee arthroplasty procedures (THA and TKA, respectively) realization in central Poland (Lodz Voivodeship). According to the Information System of the National Health Fund (SINFZ), in that period, a total of 989 THA and TKA procedures were performed in this medical facility (555 THA and 434 THA procedures). Practical experience in the use of telemedicine technologies in this facility, namely video consultations between medical specialists and patients, has been recognized as a valuable source of information about the technology market, especially because of the practical application that already takes place in the clinical activities of the facility. Presentations and materials on new technologies provided by technology suppliers and presented in the period of 2020–2021 to the medical and high administrative professionals of Medical Magnus Clinic were also taken into account in the market analysis. To this source of information, two specialistic meetings, which took place with authors at the facility, were taken into account: telerehabilitation platform and activity wristband with telemonitoring function.

## 3. Results

### 3.1. Study Identification

**Figure 3:**
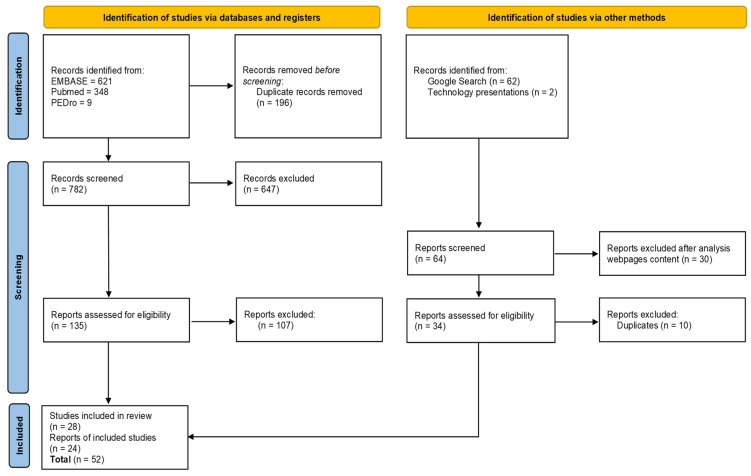
PRISMA flowchart.

After completion of the initial searches, the results were manually filtered to remove duplicates. Two independent reviewers (A.R.-S. and K.K.) then screened journal titles and abstracts for relevance. From databases, a total of 978 studies were identified through the literature search: 621 in EMBASE, 348 in PubMed, and 9 in the PEDro database. After duplicates, we screened 782 articles, and 28 articles met the final inclusion criteria (Figure 3. PRISMA flowchart; Table 1). To prepare the results, we used the PRISMA 2020 statement, which consists of a 27-item checklist (Supplementary Materials File S2) and a revised flow diagram for original and updated reviews [8].

Google Search results (22 in total sources: 15 English, 7 Polish; Table 2) and technology presentations during specialistic meetings (two sources, Supplementary Materials File S3) were included in the study as gray literature. This part of the research stands for the total final search of 24 sources. Duplicates were defined as the same technology searched at a few sources. Only one source of each technology was included in the study. In total, 52 studies were finally selected for research.

Described methods were used for the effective selection of up-to-date technologies for the posthospital patient care process after THA by particular users. The following sections of the article present the results of the research.

### 3.2. Users of Telemedicine Technologies in Posthospital Patient Care Process after THA

The users of telemedicine technologies in posthospital patient care process after THA are patients (at every stage of the process), physicians [13,14,23,24,38], physiotherapists [15,16,24,25,26], surgeons, [17,18,25], surgeon’s administrative assistants [17], health care team [27], and rehabilitation center [28].

Depending on the medical area: physiotherapy, outpatient specialist care, or telemonitoring, various users are going to be involved in the management and realization of the designed patient care process. Defining the users based on a literature search gave a clear and wide overview of the complexity of the designed process. In addition, there is a need for IT specialists and administrative staff. Users can communicate in real-time or/and on time planned via electronic technology with a delay depending on the care area of the process.

Our research team also defined the activity of the so-called patient coordinator role. It is required for the effectiveness of the process and for maintaining the patient’s sense of safety and comfort during posthospital care. Among the tasks of the coordinator would be arranging appointments, examinations, consultations, reminding the patient over the phone about appointments, reporting collected data, as well as collecting data on services provided outside the telemedicine coordinating center and related to the treatment process of a patient undergoing hip arthroplasty.

### 3.3. Data Collected from Telemedicine Technologies Used in Posthospital Patient Care Process after THA

Telemedicine connects professionals and patients through many innovative media solutions and devices [68]. The key point in data collection from telemedicine technologies is the real-time character of this process based on bi-directional online transfer. Data are collected on various levels of communication: (1) patient ↔ specialist, (2) patient ↔ guardian, (3) patient ↔ system using artificial intelligence (AI). Two types of data should be distinguished: (1) qualitative and (2) quantitative data. In the designed posthospital patient care process after THA following data are transferred over the distance: personal data, electronic medical documentation, files with medical tests results, measurement results from wearable devices (e.g., basic life parameters, parameters, and pictures of the exercises performed, gait analysis, number of steps, percentage of physical, cognitive performance, number and time of patient fall), with the use of written data, sound, images, and movies (Table 3).

### 3.4. Telemedicine Technology’s Needs in the Posthospital Patient Care Process after Total Hip Arthroplasty

Telerehabilitation, a complementary treatment to standard physical therapy, generates a positive effect on mobility in people following hip surgery [69]. Innovative technologies are proposed here to be used as elements in building telerehabilitation programs outside the medical facility during the first year after the surgery, to conduct remote patient-specialist consultations, and as constant telemonitoring of basic activity parameters [70] (Table 3).

Telemedicine technologies fulfill specific requirements and functions. We defined the functional criteria of telemedicine technologies division needed in our research process after THA (Table 4).

One of the major complications of total hip arthroplasty (THA) is prosthetic dislocation. It is a clinical practice that, to prevent early dislocation, patients are instructed about movement limitations. One of the interesting concepts is the HipDas system, which aims to accelerate the recovery of THA patients. This system supported the correct application of postoperative restraints to prevent dislocation. This project sought to gain insight into the limitations of movement that are said to have low levels of self-efficacy in daily activities, as well as to reveal the design needs for an outpatient hip dislocation warning system (HipDas) and to test its utility among patients [71]. With respect to the means by which telerehabilitation is implemented, the most frequently studied is the use of the mobile telephone with its messaging services and telephone calls, with knowledge emerging of the effectiveness of the applications available on smartphones as a digital practice tool [19].

Telemedicine technologies can be divided into two types as: (1) visual telemedicine technologies and (2) wearable telemedicine technologies. The technology users’ needs can be fulfilled through the use of correctly chosen wearable and visual telemedicine technologies in the posthospital patient care process after THA. Both types of technologies are characterized in the following article subsections.

### 3.5. Analysis of Visual Telemedicine Technologies in the Posthospital Patient Care Process after THA

Systems of remote communication with patients and online telerehabilitation are becoming more and more popular among doctors and physiotherapists due to the possibility of using them as effective therapeutic tools in the field of home rehabilitation. These systems are in different phases of research and development. Telemonitoring appears to maximize patient care and the effectiveness of treatment. The number of publications illustrates the growing interest in the matter. Telemonitoring has yet to be evaluated in the setting of postoperative care and surgical pathologies [14]. Remote monitoring and control of the performance of ordered exercises, low costs, and easy operation of the systems result in the high dynamics of the development of these tools.

To visual technologies belong, i.a.:

- Specialistic orthopedic telerehabilitation systems [20,57,58,59,60,61,62,72],
- Telerehabilitation systems using the Wii balance platform dedicated to games [73,74,75,76],
- Telerehabilitation application using smartphone camera [77],
- Technology-based home exercise program using iPad application [29],
- eHealth education program with web-based access to resources and communication log with hospital professionals [27],
- Tablet-based technology for daily biophysical measurements, photographs of operation wounds, and virtual interaction with a nurse [13],
- Mobile health applications for self-management rehabilitation intervention [30,31],
- Software for medical video consultations [15,44,45,46,47,48,49,50,53,54,55,56,78,79], some also with real-time language translation [51,52].

It is important to mention that telephone consultations are also a widely used telemedical form of communication [18].

### 3.6. Analysis of Wearable Telemedicine Technologies in the Posthospital Patient Care Process after THA

Smart-wearable devices which monitor human activity are being more and more widely used. This type of device with integrated sensors and often connected with monitoring e-centers is being popularized as internet-related technologies are in a rapid growth phase [80,81]. The need for the development and implementation of application-based technologies in ambulatory monitoring medical procedures increases every year [82,83,84,85,86], and it is also important to explore and customize technologies to the patients’ and specialists’ acceptance and perception [87]. The new approach to patient–specialist contact in a pandemic and builds a novel field for innovative technologies use in regard to remote access to quality health parameters measurement.

Among the most crucial advantages of smart wearable systems are the possibility of remote monitoring of multiple life functions, easy patient localization, ease of use, and compact form. Smart-wearable devices are useful for health promotion and have a positive impact on quality of life [88]. Wearable technologies that are available and applied to use in medicine are telemetric wrist bands with the possibility of telemonitoring service:

- Comarch telemedicine wrist band [63],
- SiDLY telemedicine wrist band [64],
- Telecare24 telecare system [65],
- MobiCare telemonitoring system [66],
- Novama Life Band telemedicine wrist band [67].

In relation to telemetric wristbands, there are the following functionalities possible:

- Fall sensor,
- GPS location,
- Activity sensor,
- Notification to take an exercise,
- Device insertion sensor,
- Defining the boundaries of the movement area (virtual fences),
- Communication by e-mail or phone to the patient’s guardian,
- 24-h monitoring center service,
- Pulse,
- Reminder for medications or fluid intake.

The presented selection shows current technological possibilities. Our study indicates that the functionality of the aforementioned wearable technologies shows the potential matching with the technological needs revealed in our originally designed process of patient care after THA. In the future, telemedicine technologies will develop faster and faster, and at the same time, the possibility of their implementation in medical procedures will increase. The high potential of telecommunication systems development is noteworthy, in particular, 5G technology [89,90,91] and solutions included in the Internet of Things (IoT) [92,93,94].

## 4. Discussion

The conducted search was comprehensive of 978 records from databases and 64 from gray literature. We identified and included fifty-three papers. The number of papers published during the last decade, especially studies published in the last 5 years, suggests that the telemedicine technologies used in posthospital patient care after total hip replacement are growing rapidly. The results indicate that telemedicine can be of value in the fast-track treatment of patients undergoing total hip replacement [26]. Telerehabilitation solutions can be delivered to patients with total hip replacement in their own homes while maintaining high levels of satisfaction. More importantly, telerehabilitation patients achieve no worse physical and functional outcomes than patients receiving in-person rehabilitation programs [19,29,95].

OHTAC (Ontario Health Technology Advisory Committee) recommendations from 2014 for patient care after THA indicate that the health system should support the move to community-based physiotherapy and discharge from acute care. A large randomized controlled trial of high quality showed that there is no advantage to receiving inpatient physiotherapy in comparison to a home-based physiotherapy program for patients after primary THA. This showed that a self-managed home exercise program with a physiotherapist monitoring/supporting phone calls as an element of remote control could be considered instead of attending outpatient physiotherapy [96]. Those recommendations show that alternative solutions in physiotherapy should be considered, including telemedicine technology. After hip arthroplasty, most patients are able to undergo rehabilitation at home, so patients should have the possibility to conduct an exercise at home and be motivated to exercise at the same time. The health care system should support rehabilitation in the patient’s home if there are indications for outpatient rehabilitation. The referral for rehabilitation should contain information on the patient’s health condition and the post-operative course of treatment and should specify, i.a., mobility restrictions. Remote home monitoring via mHealth is feasible, adaptable, and may even promote more effective postoperative care. Given the rapid expansion of mHealth, physicians and policymakers need to understand these technologies better so that they can be integrated into high-quality clinical care [38].

Our study finds that posthospital care for patient care after THA can be performed by services over telecommunication networks and the Internet, telephone calls, telephone/Internet text/image messages, synchronous videoconferencing and asynchronous multimedia online, etc. Scientific reports and technology market research show that the sector of telemedicine technologies is dynamically growing and in the field of high interest to government institutions, health organizations, scientists, technology producers, and discoverers of technology innovations. Technology market analysis showed the availability of solutions in three telemedicine areas important for the process under our study, which are teleconsultation, telerehabilitation, and telemonitoring.

Telerehabilitation solutions can facilitate access and observance of health recommendations, reduce costs [21,32,33,34], and also contribute to social distancing when it becomes necessary as an infection control action, especially during a pandemic [23]. Karlon et al. [69] found that a 6-week telerehabilitation program based on video clips of exercises added to physical therapy sessions was more effective for the recovery of physical function compared to standard rehabilitation. Dias Correia et al. [97] also showed that an 8-week telerehabilitation program was associated with better outcomes than standard rehabilitation.

We need to emphasize that systems developed for the supervising of home-based telerehabilitation programs are diverse. However, Hosseiniravandi et al. revealed that some systems have common functionalities, for example, report/statistics generating, exercise plan management, patient education, and task scheduling [22].

mHealth apps influence earlier discharge, improve patient engagement, and offer a functional for early identification of complications. Deep analysis of usability is critical to the adoption of these tools in the postoperative period [33].

As presented in the literature, patients prefer telephone calls and video consultations; however, the physical therapist must take into account the need for feedback and visual participation when utilizing telerehabilitation services [18].

Some technological barriers, ethical and legal regulations, health insurance coverage, and cultural difficulties were found [39]. The development of telemedicine is at various levels of advancement all around the world. Research shows that the possibilities of telehealth aspect growth are huge but slowed down due to various barriers. The conditions and prospects for the technological development of telemedicine lie, among others, in access to smart devices, 5G network, and the Internet of Medical Things (IoMT), as well as many others [40,41,42,70]. To decrease the barriers to entry in privacy regulations and patient access to technology, concerted efforts are needed [35,43].

Two articles that we included in our review indicate that future research studies, notably randomized controlled trials, with a well-defined research protocol are needed to demonstrate the safety and long-term efficacy of innovative medical solutions, especially for post total hip replacement rehabilitation [36,37]. Technologies based on virtual reality are promising in view of the range of motion and pain management [98,99,100], but future research is needed to determine the effectiveness of virtual reality in the rehabilitation of osteoarthritis, regarding rehabilitation after THA.

Even though telemedicine technologies are characterized by growing popularity of use and bring benefits to their users, there is still no standardized legal framework of global standards for telemedicine [101]. Attempts are being made to standardize some telemedicine procedures by spreading the so-called good practice guidelines in telemedicine [102]. Legal concerns on telemedicine technologies usage are being raised, which shows the high need for intense work in this area by government institutions and specialized legal bodies [103].

### Strengths and Limitations

The review was prepared according to PRISMA guidelines. The authors analyzed a large number of variables, allowing for a comprehensive characterization of the telemonitoring literature in the past 10 years, but some relevant earlier publications may have been missed; however, medical care technology has developed rapidly in recent years, and the pace of change has accelerated with the onset of the COVID-19 pandemic in 2020. Specifically, the gray literature was searched thoroughly. The systematic review was performed in the three most popular databases. The inclusion of only articles written in English could contribute to a publication bias. To the authors’ knowledge, no other review of this scale has been conducted.

## 5. Conclusions

In the field of telerehabilitation systems, there is a great variety of non-structured, single, non-integrated solutions, which proves the need for continuous development, giving an interesting field for research and innovation for scientists, and clinical and management specialists.

The ongoing analysis of scientific reports and research on the current technological possibilities in the market of telemedicine devices, software, and services seem to be a very important combination of forces for effective practical implementation in the processes of patient care after medical procedures. Adaptation of the set of technologies and the flow of information between technologies is a big challenge for the teams planning and implementing modern processes of care for patients after surgery.

Our research analysis and practical experience from medical facilities show that, in implementation projects, the most effective care can be obtained from the combined use of both types of technologies, wearable and visual together. We have observed the high availability of medical teleconsultations, including, importantly, video calls with electronic medical documentation, which we designed in the study process. There is also a selection of professional telemonitoring technologies, including physical activity and falls in patients.

**Figure 4:**
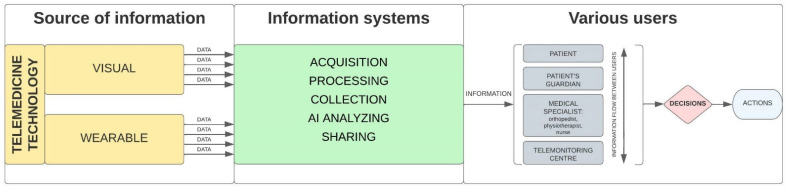
Diagram of detailed information flow in posthospital process of patient care after THA using telemedicine technologies.

Information flow logistics of patient’s health data obtained and generated from telemedicine technologies and their management are the most important to make decisions and then appropriate actions by various users in patient care after THA treatment (Figure 4). As the needs of stakeholders in the posthospital patient care process after THA differ, the awareness of appropriate telemedicine technologies selection, information flow and its management importance are prerequisites for effective patient care.

## Figures and Tables

**Table 1:** Database search results.

		Ref.
**Technological area**	Teleconsultation	[13,14,15,16,17,18,19,20,21,22]
	Telerehabilitation	[15,16,19,20,21,22,23,24,25,26,27,28,29,30,31,32,33,34,35,36,37]
	Telemonitoring	[13,14,15,20,22,30,31,33,35,38,39,40,41,42,43]
**Stakeholders in the care process**	Family physician	[20]
	Health care team	[27]
	Hospital surgical team	[20]
	Nurse	[13]
	Occupational therapist	[21]
	Orthopedic surgeon	[17,18,25]
	Patient	[13,14,15,16,17,18,19,20,21,22,23,24,25,26,27,28,29,30,31,32,33,34,35,36,37,38,43]
	Physician	[13,24,38]
	Physiotherapist	[15,16,19,22,23,24,25,26,28,34,43]
	Policymaker	[25]
	Primary caregiver	[21]
	Rehabilitation center	[28]
	Social worker	[20]
	Surgeon’s administrative assistant	[17]

**Table 2:** Google Search results by research phrase.

Google Search Phrase	Results	Language of Search
“Software for medical video consultation”	[44,45,46,47,48,49,50,51,52]	EN
	[53,54,55,56]	PL
“Software for orthopedic telerehabilitation’	[57,58,59,60,61]	EN
	[62]	PL
“Telemetric wrist band with collapse function”	[63,64]	EN
“Telecare monitoring”	[65,66]	EN
	[67]	PL

**Table 3:** Technological needs identified in the process of posthospital patient care after THA.

Medical Area	Innovation	Data Type and Usage	Telemedicine Technology Proposed by Our Research Team
Physiotherapy	Telerehabilitation program for remote self-rehabilitation at patient’s home	Qualitative and quantitative data. Personal data, electronic health records (EHR), files with medical tests results, measurement results from wearable devices (e.g., basic life parameters, parameters and pictures of the exercises performed, gait analysis, number of steps, percentage of physical, cognitive performance, number and time of patient fall), with the use of written data, sound, images, and movie. Data can be gathered and analyzed in real-time by medical specialists or/and can be analyzed by AI, compared with algorithms and automatic feedback given to the patient.	Telerehabilitation systems using AI technology in wearable and visual technologies collecting, processing, and presenting data from physical activities which were ordered by doctors and physiotherapists; system enabling physiotherapists to control and moderate ordered activities and monitoring the results.
Outpatient specialist care	Remote medical consultations with specialist personnel: nurse, doctor, physiotherapist	Qualitative and quantitative data. Personal data, electronic health records (EHR), files with medical tests results, with the use of written data, sound, online or recorded movie. Data can be gathered and analyzed real-time or/and can be analyzed by AI, compared with algorithms and automatic feedback given to the patient.	Software, application for conducting remote medical patient–specialist video-consultation with keeping EHR.
Telemonitoring	Telecare wrist band with collapse and activity sensor	Qualitative and quantitative data. Personal data, electronic health records (EHR), measurement results from wearable devices as basic life parameters, parameters and pictures of the exercises performed, gait analysis, number of steps, percentage of physical, cognitive performance, number and time of patient fall, with the use of written data (numbers, alerts, communicates) via sound and database files send to monitoring system. Data can be gathered and analyzed real-time or/and can be analyzed by AI, compared with algorithms and automatic feedback given to the patient.	Telemetric wrist bands with connection to medical centers monitoring patient’s condition and with alarm system to patient’s guardian.

Yellow color used in Table 3 corresponds to the color scheme used in Figure 1 in order to intuitively visualize the area of innovation in the process.

**Table 4:** Functional criteria of telemedicine technologies division need in the process of posthospital patient care after THA.

Technology Function	Technology Example
Obtaining information	Measuring sensors, video cameras, radars, and data collecting sensors (wearable and visual).
Information processing	Artificial Intelligence (AI) technologies, where as a result, we obtain a statement and indication of, i.e., image recognition, critical points, good/bad results, repeated errors, and progress. Medical software, applications, databases appropriate for safe and easy electronic medical data management.
Presenting information	Wireless and internet communication systems and applications using, i.a., charts, summaries, alerts including light signaling in wireless and internet communication systems including sound and light signaling, individually designed to the technology recipient/user needs and perception possibilities: to patient, to patient’s guardian, to medical doctor, to physiotherapist, to nurse, telemonitoring center staff.
Communication	Software and applications using secure IT connection and databases for remote patient-specialist medical consultations.

## Data Availability

The data presented in this study are available on request from the corresponding author.

## Supplementary Materials

The following supporting information can be downloaded at: https://www.mdpi.com/article/10.3390/ijerph191811521/s1, File S1: Database search strategy; File S2: PRISMA 2020 Checklist; File S3: Meeting summary sheet protocols.
