# Supplementary material for: Telemedicine Technologies Selection for the Posthospital Patient Care Process after Total Hip Arthroplasty
Source: Int J Environ Res Public Health. 2022 Sep 13;19(18):11521. doi: 10.3390/ijerph191811521 (PMC9517262; doi:10.3390/ijerph191811521)
Supplement: Supplementary file 1 [file ijerph-19-11521-s001.zip › File S1.pdf]

File S1 - Database search strategy

Database: **Embase** <1974 to 2022 Week 17>
Search Strategy:
----

1 exp telehealth/ or (e?health or tele?health).ti,ab,kf. or exp telemedicine/ or tele?medicine.ti,ab,kf. or telerehabilitation/ or ((e-rehabilitation or remote rehabilitation or tele?rehabilitation or virtual) adj2 rehabilitation).ti,ab,kf. or telemonitoring/ or (tele?monitoring or distant patient monitoring or remote patient monitoring).ti,ab,kf. or teleconsultation/ or ((remote consultation or tele-consultation or telephone) adj6 consultation).ti,ab,kf. or exp videoconferencing/ or (((videoconference or video) adj3 conference) or video conferencing).ti,ab,kf. (82128)
2 exp hip replacement/ or (hip replacement or hip replacement arthroplasty or hip joint replacement$1).ti,ab,kf. or exp total hip replacement/ or (total hip replacement or total replacement hip arthroplasty or hip total replacement arthroplasty or hip total arthroplasty).ti,ab,kf. or exp hip/ or hip joint$1.ti,ab,kf. or exp hip osteoarthritis/ or (hip arthrosis or hip joint arthrosis or hip osteo?arthritis or hip osteo?arthrosis or hip osteoarthrosis).ti,ab,kf. or exp hip prosthesis/ or (hip prosthes$2 or hip joint prosthesis or hip joint prosthesis or hip prostheses or hip implant).ti,ab,kf. or exp postoperative care/ or (postoperative care or postoperative therap$3 or postoperative treatment$1).ti,ab,kf. (275999)
3 "Preferred Reporting Items for Systematic Reviews and Meta-Analyses"/ or "systematic review"/ or systematic review.ti. or randomized controlled trial/ or clinical trial/ or (clinical adj1 trial*).ti,ab,kf. or systematic search*.ti,ab,kf. (2136518)
4 and/1-2 (621)
5 and/3-4 (126)
6 conference.pt. (5121210)
7 5 not 6 (107)
8 limit 7 to yr="2012 - 2022" (100)

File S1 - Database search strategy

Database: **PUBMED**
Search Strategy:

"Hip Joint/surgery"[mh] OR "Hip Joint"[mh] OR"hip joint*"[tiab] OR "arthroplasty, replacement, hip"[mh] OR "hip replacement*"[tiab] OR „Hip Prosthesis Implantation"[tiab] OR „Hip Replacement Arthroplast"[tiab] OR "Total Hip Replacement*"[tiab] OR "Total Hip Arthroplasty"[tiab] OR "osteoarthritis, hip"[mh] OR "hip osteoarthritis"[tiab] OR Coxarthrosis[tiab] OR Coxarthroses[tiab]

AND

Telerehabilitation[mh] OR Telerehabilitation*[tiab] OR "Tele-rehabilitation*"[tiab] OR "Remote Rehabilitation*"[tiab] OR "Virtual Rehabilitation*"[tiab] OR telemedicine[mh] OR telemedicine[tiab] OR „Mobile Health"[tiab] OR mHealth[tiab] OR Telehealth[tiab] OR eHealth[tiab] OR "remote consultation*"[tiab] OR "teleconsultation*"[tiab] OR "distance counseling"[mh] OR "distance counsel*"[tiab] OR "E-Therapy"[tiab] OR "E Therapy"[tiab] OR "E-Counseling"[tiab] OR "E Counseling"[tiab] OR "ehealth"[tiab] OR "telerehabilitation"[tiab] OR "telemonitoring"[tiab] OR telerehabilitation[mh] OR "Videoconferencing"[mh] OR "Remote Consultation"[mh] OR "telemedicine"[mh] OR "teleconsultation"[tiab] OR "telehealth"[tiab]

All strategy:
(Telerehabilitation[mh] OR Telerehabilitation*[tiab] OR "Tele-rehabilitation*"[tiab] OR "Remote Rehabilitation*"[tiab] OR "Virtual Rehabilitation*"[tiab] OR telemedicine[mh] OR telemedicine[tiab] OR „Mobile Health"[tiab] OR mHealth[tiab] OR Telehealth[tiab] OR eHealth[tiab] OR "remote consultation*"[tiab] OR "teleconsultation*"[tiab] OR "distance counseling"[mh] OR "distance counsel*"[tiab] OR "E-Therapy"[tiab] OR "E Therapy"[tiab] OR "E-Counseling"[tiab] OR "E Counseling"[tiab] OR "ehealth"[tiab] OR "telerehabilitation"[tiab] OR "telemonitoring"[tiab] OR telerehabilitation[mh] OR "Videoconferencing"[mh] OR "Remote Consultation"[mh] OR "telemedicine"[mh] OR "teleconsultation"[tiab] OR "telehealth"[tiab]) AND (("Hip Joint/surgery"[mh] OR "Hip Joint"[mh] OR"hip joint*"[tiab] OR "arthroplasty, replacement, hip"[mh] OR "hip replacement*"[tiab] OR „Hip Prosthesis Implantation"[tiab] OR „Hip Replacement Arthroplast"[tiab] OR "Total Hip Replacement*"[tiab] OR "Total Hip Arthroplasty"[tiab] OR "osteoarthritis, hip"[mh] OR "hip osteoarthritis"[tiab] OR Coxarthrosis[tiab] OR Coxarthroses[tiab])) Sort by: Publication Date

File S1 - Database search strategy

Database: **PEDro**
Search Strategy:

Table S1. Search strategy at PEDro database.

1st word	2nd word	Results
ehealth	**total hip arthroplasty**	1
telemedicine		0
telerehabilitation		2
telemonitoring		0
teleconsultation		0
remote consultation		0
ehealth	**hip replacement**	0
telemedicine		0
telerehabilitation		4
telemonitoring		0
teleconsultation		0
remote consultation		0
ehealth	**hip joint**	0
telemedicine		0
telerehabilitation		0
telemonitoring		0
teleconsultation		0
remote consultation		0
ehealth	**hip osteoarthritis**	1
telemedicine		0
telerehabilitation		3
telemonitoring		0
teleconsultation		0
remote consultation		0
ehealth	**postoperative care**	2
telemedicine		0
telerehabilitation		2
telemonitoring		0
teleconsultation		0
remote consultation		0
