# Supplementary material for: Telemedicine Technologies Selection for the Posthospital Patient Care Process after Total Hip Arthroplasty
Source: Int J Environ Res Public Health. 2022 Sep 13;19(18):11521. doi: 10.3390/ijerph191811521 (PMC9517262; doi:10.3390/ijerph191811521)
Supplement: Supplementary file 1 [file ijerph-19-11521-s001.zip › File S3.pdf]

File S3 - Meeting summary sheet protocols

Table S3. Meeting summary sheet. Sheet code: A/2022.

Date/week day	Place - address	Start time	End time	Duration
15th March 2021<br>Monday	Medical Magnus Clinic<br>Lodz, Poland	16:00	20:00	4 h

1. **Presenting company/brand:** RehaBee Application – telerehabilitation application for home use by patients. Polish brand of application for telerehabilitation with mobile phone camera use.
2. **Topic of the meeting:** Telerehabilitation technology presentation and analyze of possibility of application use for facility patients after orthopedic surgeries.
3. **Form of the meeting:** *Personal at the medical facility, Medical Magnus Clinic in Lodz, Kopernika 38 Street, Poland*
4. **Participants (occupational group, function)**

1. *RehaBee CEO presenting the functionality and business use profile of the tool*
2. *RehaBee Engineer presenting the functionality of the tool, Business Analyst, UX Designer*
3. *Researcher - Orthoapedist – Medical Magnus Clinic CEO, medical doctor with over 40 years' experience on orthopedy*
4. *Researcher - Physiotherapist – Medical Magnus Clinic substantive consultant of the rehabilitation department*
5. *Researcher - Public Health Specialist - Medical Magnus Clinic Marketing Director, specialist with 14 years' experience on medical devices market and health sector*

6. **Source of data:**

Personal presentation of application use at the facility with practical showing of app work.
Producers product webpage www.rehabee.pl

Remarks on technology:

Telerehabilitation application/platform for patient, physiotherapist and medical doctor us. Patients can use it at home. The technology use mobile phone camera for view transmission (important as it does not require high-tech equipment to work). Software enables the medical specialist to set up individual or standard rehabilitation program, measuring the results obtained in real-time and after.
Application is still under development and looking for investors to buy//use the technology, to enlarge the base of algorithms used.
Sum-up: easy to use app for patient's home self-telerehabilitation. Uses 2D technology, suggested considering 3D. Using mobile phone camera for visual contact and recording the exercises, movement correction is advantageous feature. The tool may be useful for an overall assessment of the patient's activity after surgery.

Karolina Kamecka

Researcher's signature

Appendix C - Meeting summary sheet protocols

Table D2. Meeting summary sheet. Sheet code: B/2022.

Date/week day	Place - address	Start time	End time	Duration
28nd September 2020<br>Tuesday	Medical Magnus Clinic<br>Lodz, Poland	10:00	12:30	02:30 h

1. **Presenting company/brand:** Comarch – international IT company offering Hospital Information System for electronic medical records; telemedicine devices..

5. **Topic of the meeting:** Presentation of Comarch Healthcare S.A. solutions with particular emphasis on telecare solutions.

6. **Form of the meeting:** *Online* meeting with the COMARCH Business Development Manager and researchers group

7. **Participants (occupational group, function)**

   1. *Business Development Manager – Comarch*
   2. *Researcher - Expert at logistics and management, telematics, dissertation supervisor, professor at medical university*
   3. *Researcher - Public Health Specialist – doctoral student, Medical Magnus Clinic Marketing Director, specialist with 14 years' experience on medical devices market and health sector*

7. **Source of data:**

Online detailed presentation of telecare solutions.

Producers product webpage https://www.comarch.com/iot-ecosystem/internet-of-healthcare-things/

Remarks on technology:

Producer has in the offer a wide selection of telecare devices and telemedicine software, as well as telemonitoring function of the devices. In the offer there is already used in few local and nationwide in regards to alarm/life/collapse wrist bands. Telemonitoring function is available depending on the offer range. Possible to be bought by private users and institutions. This is the best product for the offer which can be useful in patient care process after surgery. The company has offices around the world and the Life-wrist-band (so called Angels-band) is already solution used outside the Poland, example Spain The offer was presented in details and possibility of visiting medicine center of Comarch stays open for researchers.

Karolina Kamecka

Researcher's signature
